# *NFIA* haploinsufficiency: case series and literature review

**DOI:** 10.3389/fped.2023.1292654

**Published:** 2023-10-17

**Authors:** Gianluca Dini, Alberto Verrotti, Paolo Gorello, Luca Soliani, Duccio Maria Cordelli, Vincenzo Antona, Amedea Mencarelli, Davide Colavito, Paolo Prontera

**Affiliations:** ^1^ Department of Pediatrics, University of Perugia, Perugia, Italy; ^2^ Department of Chemistry, Biology and Biotechnology, University of Perugia, Perugia, Italy; ^3^ IRCCS Istituto Delle Scienze Neurologiche di Bologna, UOC di Neuropsichiatria Dell'Età Pediatrica, Bologna, Italy; ^4^ Dipartimento di Scienze Mediche e Chirurgiche (DIMEC), Università di Bologna, Bologna, Italy; ^5^ Department of Health Promotion, Mother and Child Care, Internal Medicine and Medical Specialties “G. D'Alessandro,” University of Palermo, Palermo, Italy; ^6^ Medical Genetics Unit, S. Maria della Misericordia Hospital, Perugia, Italy; ^7^ Research & Innovation S.R.L. (R&I Genetics), Padova, Italy

**Keywords:** *NFIA*, neurodevelopmental disorders, intellectual disability, genetics, pediatrics

## Abstract

**Background:**

*NFIA*-related disorder (OMIM #613735) is an autosomal dominant neurodevelopmental disorder characterized by a variable degree of cognitive impairment and non-specific dysmorphic features. To date, fewer than thirty patients affected by this disorder have been described.

**Methods:**

Our study included three children with *NFIA* haploinsufficiency recruited from three medical genetics centers. Clinical presentations were recorded on a standardized case report form.

**Results:**

All patients presented a variable degree of intellectual disability. None of the individuals in our cohort had urinary tract malformations. Three novel mutations, c.344G>A, c.261T>G, and c.887_888del are reported here.

**Conclusion:**

*NFIA* haploinsufficiency can be suspected through careful observation of specific dysmorphisms, including macrocephaly and craniofacial abnormalities. Instrumental tests such as MRI and renal ultrasound provide further diagnostic clues, while genetic testing can confirm the diagnosis.

## 1. Introduction

The *NFIA* (nuclear factor I/A) gene encodes a transcription factor belonging to the nuclear factor I family and plays key roles in central nervous system (CNS) development, including axonal growth, glial cell differentiation, and neuronal migration (1). Initially, the phenotypic presentation resulting from deletions affecting *NFIA* was referred to as chromosome 1p32-p31 deletion syndrome. However, the observation of overlapping phenotypes in patients with chromosome translocations that disrupt *NFIA* has demonstrated that the loss of function of *NFIA* is responsible for most of the phenotypes associated with the 1p32-p31 deletion (2–4). Haploinsufficiency of *NFIA* causes a syndrome characterized by brain malformations, most commonly abnormalities of the corpus callosum, with or without urinary tract defects (BRMUTD, OMIM#613735). Additional features include macrocephaly, seizures, developmental delay, ventriculomegaly, and hypotonia (5, 6). To date, fewer than thirty patients affected by this disorder have been described. Herein, we report three additional patients showing a variable degree of intellectual disability (ID) and CNS abnormalities, all carrying a heterozygous *de novo* mutation in the *NFIA* gene. Our findings provide further evidence that the *NFIA* gene has a role in development of the CNS.

## 2. Materials and methods

Our analysis included three molecularly confirmed *NFIA* haploinsufficiency patients, aged between 3 and 13 years, none of whom have been previously described. Genomic DNA of the patients was extracted from peripheral blood using standard methods. Information on the age and sex of the children was obtained from their medical records. DNA samples were collected as parent/child trios. Exome sequencing (ES) was performed for Patient 1 and his parents using a Twist Custom Panel (Twist Bioscience, San Francisco, CA, USA) on a NovaSeq 6,000 platform (Illumina Inc., San Diego, CA, USA). For patients 2 and 3, we performed ES combined with variant analysis within a panel of neurodevelopmental disorders-related genes, as previously described (7). All patients were born to non-consanguineous parents. The study was conducted in accordance with the Declaration of Helsinki. Written informed consent has been obtained from the parents to publish clinical information with photographs. We summarized the molecular findings and clinical presentation of our patients in Table 1.

**Table 1:** Clinical characteristics of patients in our cohort.

Feature	Patient 1	Patient 2	Patient 3
Sex and age (years)	Male, 3 years	Female, 7 years	Male, 13 years
Genotype	c.261T>G	c.344G>A	c.887_888del
Amino acid change	p.(Tyr87*)	p.(Arg115Gln)	p.(Gly296Alafs*16)
Reference sequence	NM_005595.4	NM_005595.4	NM_005595.5
Inheritance	*de novo*	*de novo*	*de novo*
Macrocephaly	Y	–	Y
Developmental delay	Y	Y	Y
IQ impairment	Y	Y	Y
Speech	*Single words*	*Articulate*	*On average*
Dysmorphic features	High forehead, short palpebral fissures, flat nasal bridge, anteverted nostrils large philtrum, macroglossia and crumpled helix.	Slight dysmorphisms	High forehead, frontal bossing, down slanting palpebral fissures, pointed chin, anteverted nostrils and crumpled helix.
Behavioral disorders	–	ADHD	–
Hypoplasia of the corpus callosum	–	Y	Y
Ventricular anomalies	–	Y	Y
Chiari I malformation	Y	–	–
Seizures	–	–	–
Urogenital anomalies	–	–	–
Skeletal abnormalities	Congenital monolateral talipes valgus	Pes planus, joint hyperlaxity	Pes planus
Cardiac defects	Tricuspid regurgitation	–	–
Hearing loss	–	–	–
Ocular findings	–	–	Convergent strabismus

ADHD, attention deficit hyperactivity disorder; Y, present.

## 3. Results

### 3.1. Patient 1

**Figure 1:**
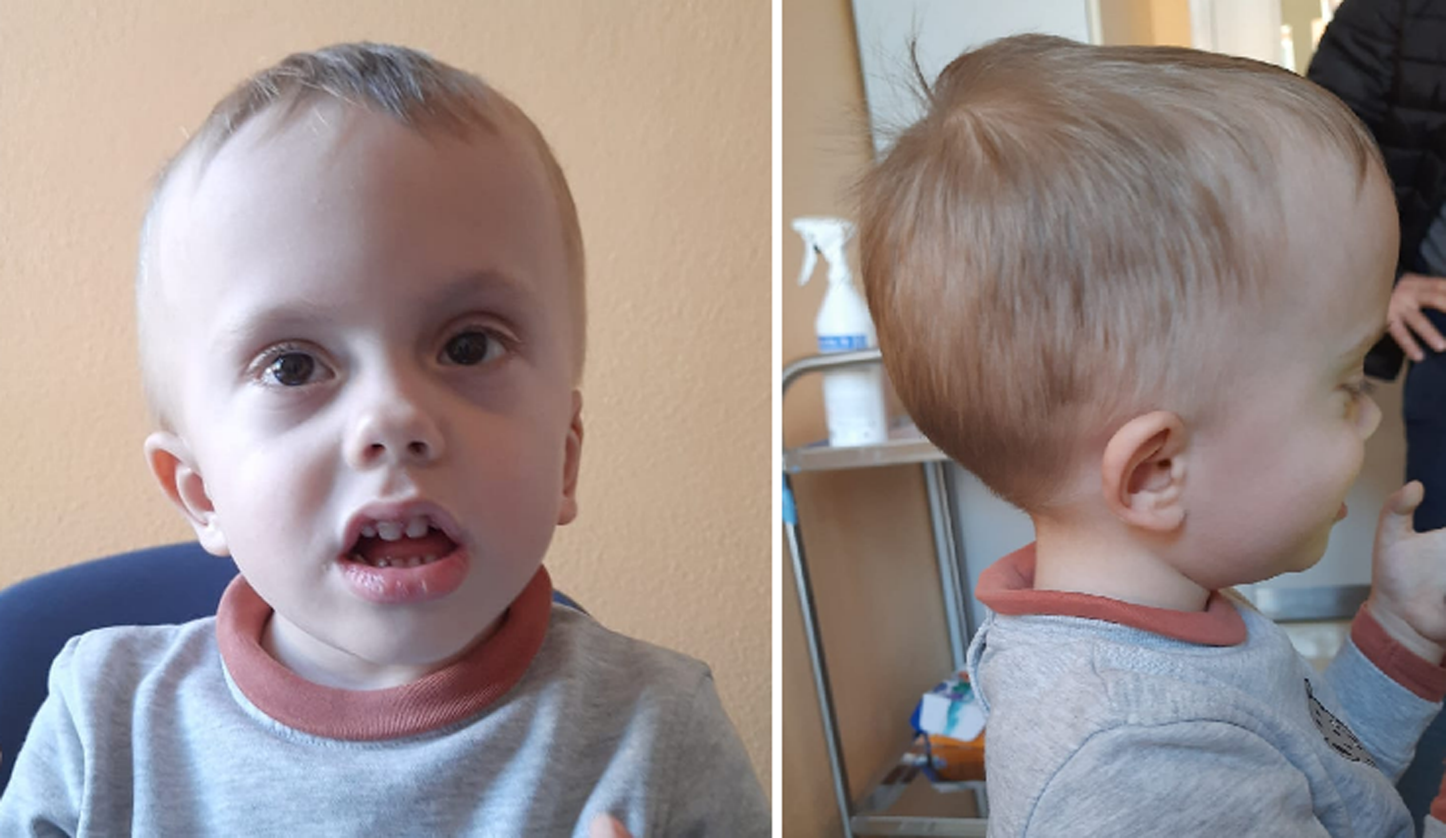
Facial features of patient 1 (c.261T>G, p.Tyr87*), including high forehead, short palpebral fissures, flat nasal bridge, and large philtrum.

**Figure 2:**
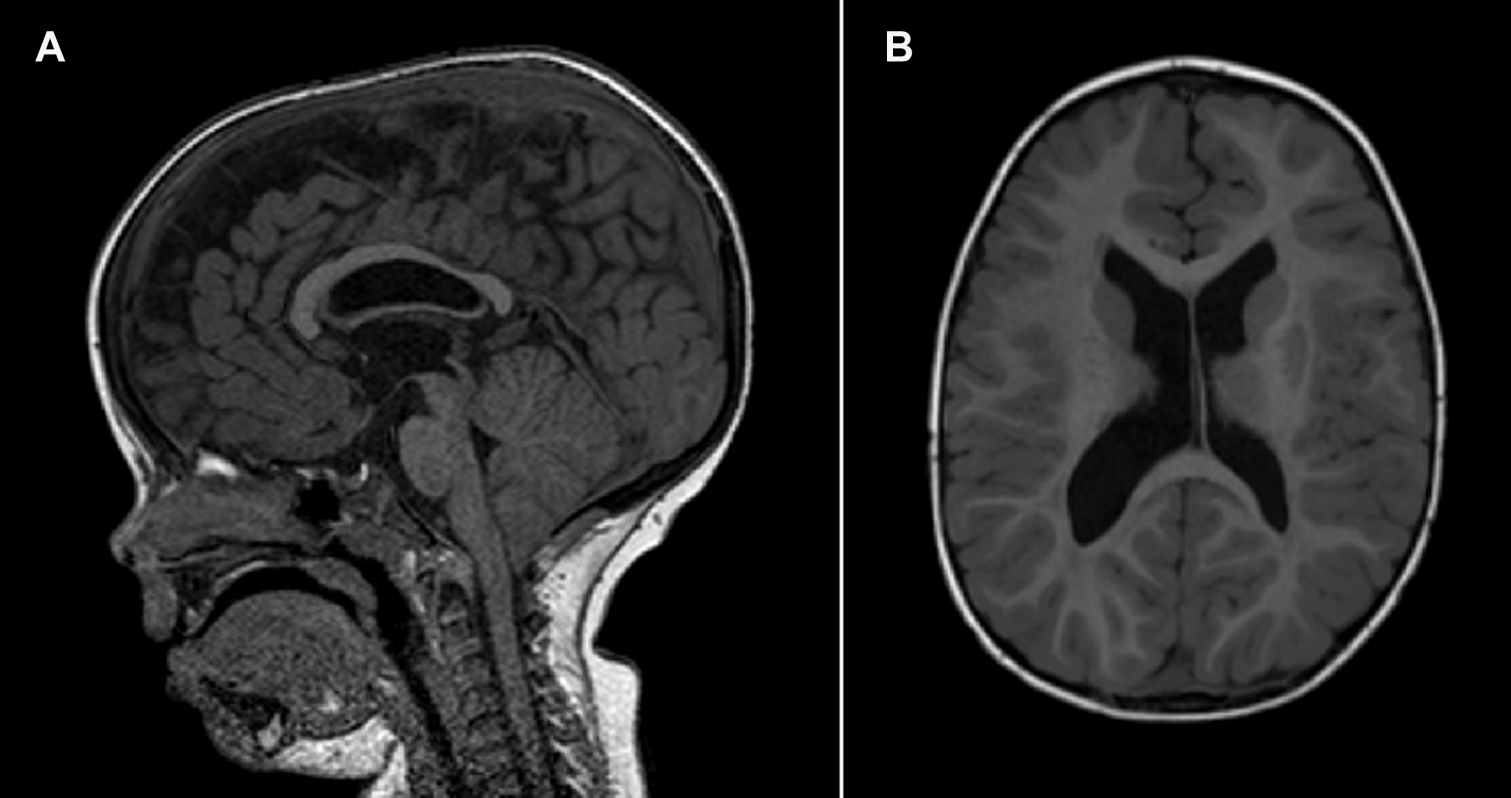
Brain MRI of patient 1 at 2.5 years of age. (**A**) Mid-sagittal T1-weighted image showing Chiari I malformation. (**B**) Axial T1-weighted image showing subependymal heterotopia.

Patient 1 (P1) is a 3-year-old male who was referred to the pediatric unit for macrocephaly and developmental delay. He was born after 39 weeks of gestation by C-section because of non-reassuring fetal status. Pregnancy was uncomplicated by teratogenic exposures or maternal illness. Birth weight was 3,620 g (76th centile), his head circumference was 39.5 cm (+4.7 SD), and his birth length was 50 cm (50th centile). Regarding his developmental milestones, he started walking without support at 15 months and speaking his first words at 19 months. The Bayley Infant Development Scale (3rd edition, BSID-III) at 2 years of age, demonstrated moderate global cognitive impairment. At the time of referral, his head circumference was 57.0 cm (>97th centile). The patient presented with a high forehead, prominent occipital protuberance, short palpebral fissures, flat nasal bridge, large philtrum, macroglossia, and a crumpled helix (Figure 1). Speech consisted of about three words in an appropriate context. The brain MRI carried out at 2.5 years, showed Chiari I malformation and subependymal grey matter heterotopia (Figure 2). Trio exome analysis revealed a *de novo* mutation c.261T>G, p.(Tyr87*) in the NFIA gene.

### 3.2. Patient 2

**Figure 3:**
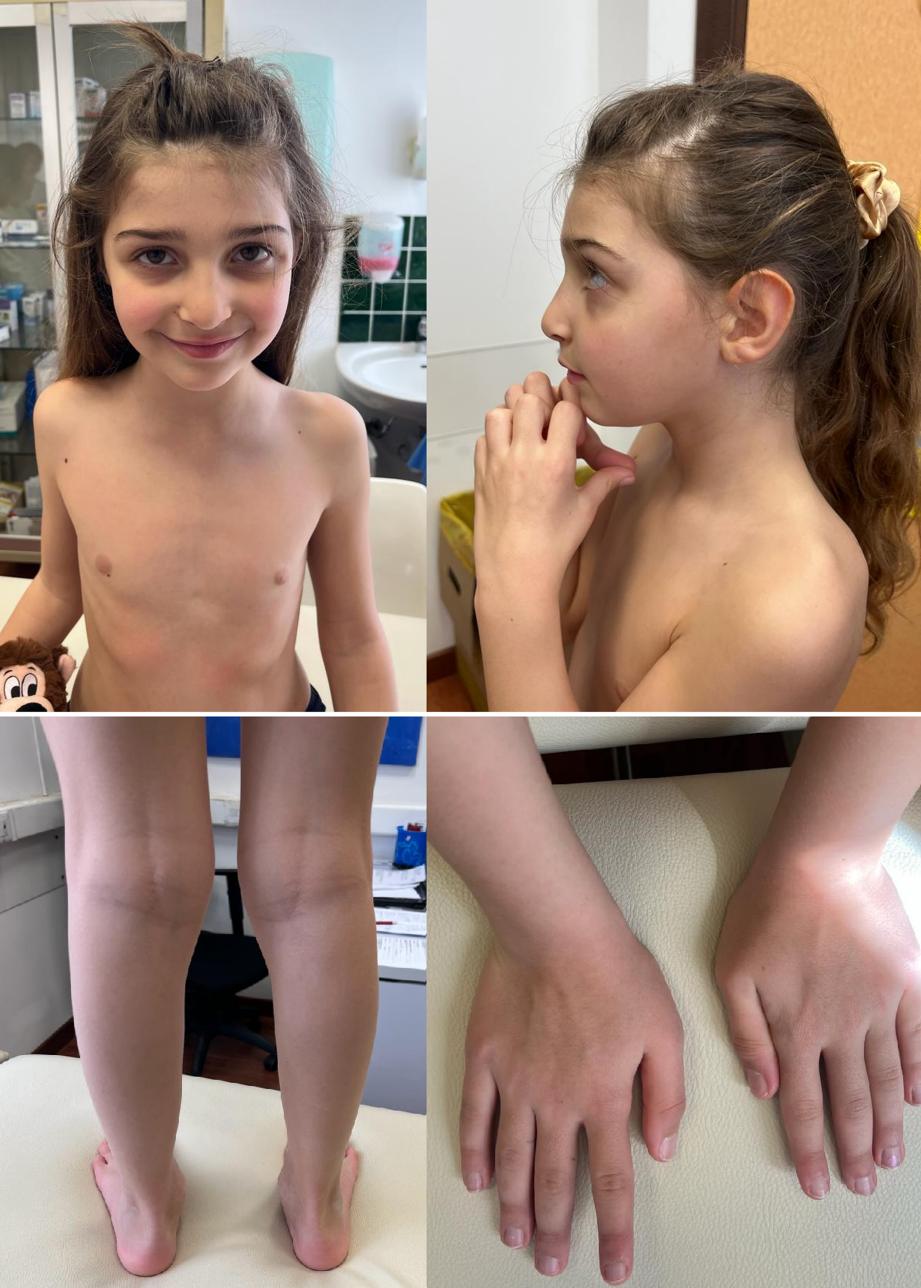
Photographs of patient 2 (c.344G>A, p.Arg115Gln) showing mild dysmorphic facial features and pes planus.

**Figure 4:**
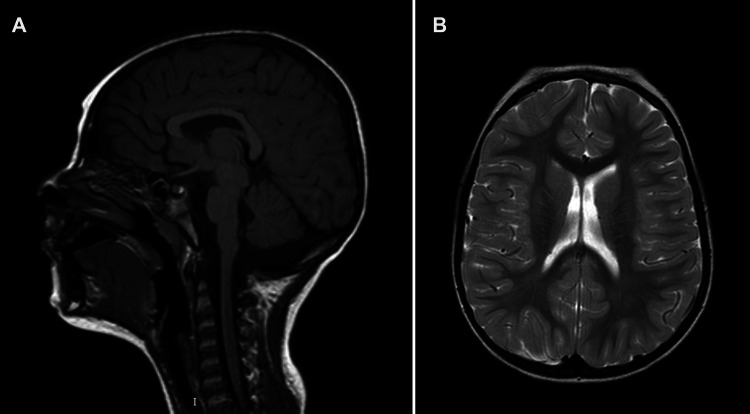
Brain MRI of patient 2 at 3 years of age. (**A**) Mid-sagittal T1-weighted image showing hypoplasia of the corpus callosum. (**B**) Axial T2-weighted image at the level of the lateral ventricles.

Patient 2 (P2) (Figure 3) is the second child of non-consanguineous parents with an unremarkable family history. She was born by vaginal delivery after an uneventful pregnancy. The birth weight was 2,945 g (12th centile), length 48 cm (14th centile), and head circumference 34.5 cm (64th centile). No apparent external malformations were observed. The brain MRI at 3 years of age (Figure 4) revealed a thin corpus callosum, dysmorphic appearance of the lateral ventricles, and a retrocerebellar arachnoid cyst. Renal ultrasound was normal. When last examined at age 7 years, her height was 134 cm (>97th centile), her weight was 28.5 kg (90th centile). Her head circumference was within the normal range, measuring at 53 cm (63rd centile). The neurobehavioral evaluation showed a learning disability and psychopathological findings, such as attention deficit hyperactivity disorder (ADHD), bizarre behaviour and emotional dysregulation. No epileptic seizures have been observed to date. Trio ES identified a heterozygous missense variant (c.344G>A, p.Arg115Gln) in the NFIA gene.

### 3.3. Patient 3

Patient 3 (P3) is the male, first child of healthy non-consanguineous parents. The family history was uneventful for disabilities. We first evaluated the patient at the age of 5.1 years for macrocephaly and developmental delay. The weight was 21.8 kg (75–90th centile), height 116 cm (95th centile), and head circumference 56.5 cm (>97th centile). Facial anomalies included frontal bossing, high forehead, down-slanting palpebral fissures, a pointed chin, anteverted nostrils, and crumpled helix in the left ear. Ophthalmologic examination, auditory brainstem response, electroencephalography, echocardiography and renal ultrasound were unremarkable. Brain MRI revealed a hypoplastic corpus callosum with enlarged ventricles and agenesis of the septum pellucidum. Metabolic work up, karyogram, chromosomal microarray, and genetic studies for Sotos syndrome were all normal. At the age of 13, the family pediatrician required new medical advice for dysmorphisms. We performed trio ES. This analysis revealed a *de novo* heterozygous frameshift mutation (c.887_888del, p.Gly296Alafs*16) in the NFIA gene.

## 4. Discussion

The *NFIA* gene belongs to a family of genes known as NFI, which also consists of *NFIB*, *NFIC*, and *NFIX* in vertebrates (8). Lu et al. (9) initially proposed the hypothesis that *NFIA* haploinsufficiency might be responsible for CNS malformations, as well as ureteral and renal defects. These authors also demonstrated ventricular enlargement, callosal agenesis, and urinary tract defects in homozygous *Nfia*^−/−^ mice and heterozygous *Nfia*^+/−^ mice. Interestingly, haploinsufficiency of the *NFIB* and *NFIX* genes also causes callosal agenesis (10–12), and missense mutations in the *NFIX* gene have been associated with Sotos-like features (10, 13).

In this case series, we described the clinical and molecular characteristics of three patients with *NFIA* haploinsufficiency. Similar to previous reports, the patients in our study presented with CNS abnormalities and a variable degree of intellectual disability. Table 2 provides a comparison of our cohort and previously reported patients with *NFIA* mutations. Hereafter, the major clinical characteristics have been grouped into macroareas.

**Table 2:** Frequency of specified features in our patients compared with cumulative data (ours and previously reported cases).

Feature	This study (3)	Zhang et al. 6 (1)	Lu et al. 9 (5)	Bertini et al. 14 (2)	Revah-Politi et al. 15 (4)	Rao et al. 16 (1)	Nyboe et al. 17 (1)	Bayat et al. 18 (2)	Negishi et al. 19 (1)	Uehara et al. 20 (2)	Mikhail et al. 21 (1)	Cumulative (total number)
DYSMORPHISMS												
Macrocephaly	2	1	-	1	4	1	1	2	1	2	1	16 (18) 88%
High forehead	2	1	-	2	-	1*	1*	2*	1	2	1	13 (14) 92%
Low-set ears	0	0	-	2	-	1*	1	2	-	0	1	7 (13) 53%
Bilateral proximally placed first fingers	0	0	-	2	1	-	1	2	-	0	-	6 (15) 40%
CNS DEFECTS												
Corpus callosum anomalies	2	1	5	2	3	1	1	2	1	2	1	21 (23) 91%
Ventricular anomalies	2	1	5	1	3	1	1	2	1	1 (1)	0	18 (22) 82%
Chiari I malformation	1	0	3 (4)	1	2	0	0	0	0	0	0	7 (22) 32%
Intellectual disability	3	-	-	2	1 (3)	1	-	2	1	1 (1)	1	12 (14) 86%
NEUROLOGICAL/ BEHAVIORAL ABNORMALITIES												
Developmental delay	3	1	5	1	4	1	1	2	1	2	-	21 (22) 95%
Seizures	0	0	3 (3)	0	3	-	-	-	0	-	1	7 (15) 47%
NDDs	1	0	-	2	1	1	-	-	-	-	-	5 (11) 45%
**URINARY TRACT DEFECTS**	0	0	3 (3)	0	1 (3)	1	0	0	1	0	0	6 (20) 30%

-: not evaluated; *: deduced by the picture; CNS: central nervous system; NDDs: neurodevelopmental disorders. The bracketed numbers denote the total number of patients in which the feature/data has been evaluated.

### 4.1. Dysmorphisms

Among the physical anomalies, macrocephaly and a high forehead are recurrent signs. In our cohort, P1 and P3 exhibited the typical facial features of *NFIA* haploinsufficiency. P2 presented a milder phenotype with only slight dysmorphisms. Craniofacial anomalies, such as asymmetries and craniosynostosis, are commonly observed in patients with *NFIA* haploinsufficiency.

### 4.2. Developmental progress/learning difficulties

The developmental milestones, especially speech, were delayed in our cohort; P2 and P3 began using single words by 2 years of age, while P1 had only a few words by the age of three. All children achieved walking. In no instances did patients lose skills or regress. All patients received special education at school.

### 4.3. CNS defects

CNS defects, most commonly abnormalities of the corpus callosum, are a prominent characteristic of *NFIA* haploinsufficiency. In addition to these, a range of less frequent and heterogeneous CNS anomalies have been reported, including cortical malformations, non-progressive ventriculomegaly, hydrocephalus, Chiari type I malformation, and tethered spinal cord.

### 4.4. Seizures

Seizures have been observed in approximately 15% of the *NFIA* patients (14). In our study, none of the patients experienced seizures. EEG was performed for P2 and P3, and the results were normal for both.

### 4.5. Neurodevelopment/behavior

Developmental delay and/or ID, ranging from very mild to severe, are invariably present in this disorder. Beside ID and developmental delay, patients may exhibit other neurodevelopmental disorders, including hyperactivity disorder and oppositional defiant disorder (ODD) (14). In our cohort, one patient (P2) had a diagnosis of ADHD.

### 4.6. Urinary trait defects

Renal/urinary defects are considered a key feature of *NFIA*-related disorder (9). The most common among these defects are vesicoureteral reflux and hydronephrosis, which can occur unilaterally or bilaterally. Other phenotypes associated with *NFIA*-related disorder include pyelonephritis, ureterovesical junction diverticulum, dysplastic kidneys, and renal cysts.

According to Revah-Politi et al., approximately 50% of patients diagnosed with *NFIA*-haploinsufficiency exhibited genitourinary defects (15). However, a subsequent literature review indicated a lower prevalence of urinary tract abnormalities (<20%). In our cohort, no patients presented with genitourinary abnormalities. Nevertheless, a surveillance of kidneys and urinary trait is recommended when *NFIA*-haploinsufficiency is detected.

### 4.7. Musculoskeletal abnormalities

Although musculoskeletal manifestations are not considered a typical sign of *NFIA*-related disorder, these features are frequently found in patients with the *NFIA* gene mutation. Bilateral proximally placed first fingers seem to be a frequently reported dysmorphism in patients with *NFIA*-related disorder (14). In our study, two patients (P2 and P3) presented with pes planus, while another with congenital talipes valgus (P1). Interestingly, it was demonstrated that *NFIA* and GATA-binding protein 3 regulate the differentiation of embryonic articular cartilage in chicken (22). The musculoskeletal manifestations associated with *NFIA* haploinsufficiency need further characterization to provide more definitive insights and treatment options for patients.

## 5. Conclusions

NFIA haploinsufficiency can be suspected through careful observation of specific dysmorphisms, including macrocephaly and craniofacial abnormalities. Instrumental tests, such as MRI and renal ultrasound, can offer further diagnostic clues, with definitive diagnosis achievable through genetic testing.

Three novel mutations, c.344G>A, c.261T>G, and c.887_888del are reported here. Like other authors, we did not identify a predictive value for specific mutations. However, the mutation c.344G>A might correlate with a milder phenotype. With the current use of clinical exome sequencing, it is expected that more patients with point mutations or microdeletions in NFIA will be diagnosed. Currently, a specific treatment for NFIA-related disorder is lacking, and symptomatic supportive treatment remains the main approach in clinical practice.

## Data Availability

The datasets presented in this study can be found in online repositories. The names of the repository/repositories and accession number(s) can be found below: https://databases.lovd.nl/shared/variants/0000933608#00014516 https://databases.lovd.nl/shared/variants/0000933609#00014516 https://databases.lovd.nl/shared/variants/0000933610#00014516
